# A review of *Salmonella enterica* with particular focus on the pathogenicity and virulence factors, host specificity and antimicrobial resistance including multidrug resistance

**DOI:** 10.14202/vetworld.2019.504-521

**Published:** 2019-04-06

**Authors:** Saleh Mohammed Jajere

**Affiliations:** 1 Department of Pathology and Microbiology, Faculty of Veterinary Medicine, Universiti Putra Malaysia, 43400 UPM Serdang, Selangor, Malaysia; 2 Department of Veterinary Public Health and Preventive Medicine, Faculty of Veterinary Medicine, University of Maiduguri, PMB 1069, Maiduguri, Borno State, Nigeria

**Keywords:** Enteritidis, foodborne pathogens, Heidelberg, multidrug-resistant, pathogenicity and virulence factors, *Salmonella enterica*, Typhimurium

## Abstract

*Salmonella* genus represents the most common foodborne pathogens frequently isolated from food-producing animals that is responsible for zoonotic infections in humans and animal species including birds. Thus, *Salmonella* infections represent a major concern to public health, animals, and food industry worldwide. *Salmonella enterica* represents the most pathogenic specie and includes > 2600 serovars characterized thus far. *Salmonella* can be transmitted to humans along the farm-to-fork continuum, commonly through contaminated foods of animal origin, namely poultry and poultry-related products (eggs), pork, fish etc. Some *Salmonella* serovars are restricted to one specific host commonly referred to as “host-restricted” whereas others have broad host spectrum known as “host-adapted” serovars. For *Salmonella* to colonize its hosts through invading, attaching, and bypassing the host’s intestinal defense mechanisms such as the gastric acid, many virulence markers and determinants have been demonstrated to play crucial role in its pathogenesis; and these factors included flagella, capsule, plasmids, adhesion systems, and type 3 secretion systems encoded on the *Salmonella* pathogenicity island (SPI)-1 and SPI-2, and other SPIs. The epidemiologically important non-typhoidal *Salmonella* (NTS) serovars linked with a high burden of foodborne *Salmonella* outbreaks in humans worldwide included Typhimurium, Enteritidis, Heidelberg, and Newport. The increased number of NTS cases reported through surveillance in recent years from the United States, Europe and low- and middle-income countries of the world suggested that the control programs targeted at reducing the contamination of food animals along the food chain have largely not been successful. Furthermore, the emergence of several clones of *Salmonella* resistant to multiple antimicrobials worldwide underscores a significant food safety hazard. In this review, we discussed on the historical background, nomenclature and taxonomy, morphological features, physical and biochemical characteristics of NTS with a particular focus on the pathogenicity and virulence factors, host specificity, transmission, and antimicrobial resistance including multidrug resistance and its surveillance.

## Introduction

*Salmonella* represents a large genus of global public health significance and is the leading cause of foodborne illnesses responsible for thousands of deaths worldwide [1-9]. *Salmonella* is Gram-negative, rod-shaped bacteria, and facultative anaerobes belonging to the family Enterobacteriaceae. The genus *Salmonella* belongs to two broad species namely *Salmonella* *enterica* and *Salmonella bongori*. So far, more than 2600 serovars belonging to *S. enterica* have been described worldwide, and many of these serovars are capable of causing illnesses in both humans and animals [10]. While few variants of *S*. *enterica* namely *Salmonella* Gallinarum (SG) and *Salmonella* Pullorum (SP) are non-flagellated and non-motile, the majority of members in the genus *Salmonella* are motile by peritrichous flagella. The SG and SP are associated with clinical disease in poultry, and they cause considerable economic losses – due to the replacement of infected flocks and associated treatment costs – to poultry farmers, especially in developing countries of the world [11-13]. In general, the genus has a predilection limited to the digestive tracts of both humans and animals hosts. Thus, the presence of *Salmonella* in other habitats such as the water, environment, and food represents fecal contamination. Recent data from the United States, European countries, and low- and middle-income countries (LMICs) indicate that *Salmonella* cases are the most commonly encountered cause of bacterial foodborne disease globally and hence supporting the fact that the control programs aimed at reducing the *Salmonella* contamination along the food chain have not been successful [14]. Consequently, there will be increased frequency and persistence of *S. enterica* in the intestinal tracts of food animals and this situation creates chronic or non-symptomatic carriers that continue to shed the organism in their feces. Thus, these carriers serve as reservoirs for future contamination and spread of *Salmonella* by contaminated milk, meat, eggs and other agricultural products fertilized and grown in *Salmonella* contaminated manure [14].

In recent years, the development of antimicrobial resistance (AMR) among foodborne pathogens such as *Salmonella* have been associated with an increased number of human deaths, longer duration of hospital stay, and high costs of treatment due to therapy failure. Several clones of multidrug-resistant (MDR) *Salmonella* have emerged during the late 1990s and early 2000s and since then, their prevalence both in humans, domestic animals and other wildlife species have expanded globally [9,15-25]. Recently, the increasing prevalence of MDR *Salmonella* such as resistance towards clinically important antimicrobials like fluoroquinolones and third-generation cephalosporins has become an emerging problem worldwide [16,18,26-33]. In recent years, studies on the global burden of non-typhoidal *Salmonella* (NTS) have shown an increasing incidence of NTS. For instance, one of these studies estimated that there are approximately 94 million cases of NTS gastroenteritis resulting in 155,000 deaths globally each year [34]. According to this study, majority of the NTS burden was found in the Southeast Asian and the Western Pacific regions [34,35].

Moreover, of the 94 million NTS cases reported [34], 80.3 million cases were estimated as foodborne origin [15]. Among the NTS, *Salmonella* Typhimurium (ST), *Salmonella* Enteritidis (SE), *Salmonella* Heidelberg (SH), and *Salmonella* Newport (SN) are the epidemiologically important NTS serotypes – with poultry and poultry derived products as important reservoir sources – and have been associated with the majority of human salmonellosis burden worldwide [5,36-40]. *S. enterica* is widely distributed in the environment and has been associated with a variety of infections in cattle, pigs, and birds including poultry and free-living wild birds [40-53].

Until today, *Salmonella* including MDR strains remains one of the leading bacterial foodborne cause of deaths especially in the LMICs [24,54]; where foods/ready-to-eat foods are prepared under less hygienic environments and fruits and vegetables are grown in farms with poor management practices. In many of these countries, these foods are sold by people who are less enlightened about the risks posed by foodborne pathogens. This review paper attempts to discuss on the historical background, nomenclature and taxonomy, morphological features, physical and biochemical characteristics of NTS with particular emphasis on the pathogenicity and virulence factors, host specificity, AMR including MDR and its surveillance.

### Brief History

The first study of *Salmonella* began during the early 19^th^ century by Eberth, who first recognized the organism and subsequently Gaffky isolated the bacillus responsible for human typhoid fever [55]. Thereafter, in 1885 Theobald Smith together with Daniel Elmer Salmon discovered and isolated *Salmonella* from the intestines of pigs infected with classical swine fever (hog cholera). During this period, they thought the bacterium was the etiological agent of hog cholera [55,56]. Later, the bacterial strain was named *Salmonella* after Dr. Daniel Elmer Salmon, an American pathologist who has worked together with Smith [57]. In recent years, the issue of nomenclature of the genus *Salmonella* has been complex, controversial, and still remains subject of debate [57]. At present, most *Salmonella* reference centers in the world including the Centers for Disease Control (CDC) adopt the nomenclatural system of *Salmonella* as recommended by the World Health Organization (WHO) [58]. This nomenclatural system classifies the genus *Salmonella* into two species based on differences in their 16S rRNA sequence analysis. These two broad species included *S. enterica* (type species) and *S. bongori* [57].

### Nomenclature/Taxonomy

On the basis of biochemical properties and genomic relatedness, the *S. enterica* is further classified into six subspecies [59]. These subspecies in the nomenclature are denoted by Roman numerals: I. *S. enterica* subsp. *enterica*; II. *S. enterica* subsp. *salamae*; III. *S. enterica* subsp. *arizonae*; IIIa. *S. enterica* subsp. *diarizonae*; IV. *S. enterica* subsp. *houtenae*; and V. *S. enterica* subsp. *indica*. Of all the subspecies of *Salmonella*, the *S. enterica* subsp. *enterica* (I) is the most common and is found predominantly associated with mammals and attributes about 99% of *Salmonella* infections in humans and warm-blooded animals. On the other hand, the other five *S. enterica* subspecies and *S. bongori* are rare in humans and are mainly found in cold-blooded animals and the environment [60].

The Kauffman and White classification system is another system in addition to the classification of subspecies based on phylogeny [61,62]. This scheme classifies *Salmonella* further into serotypes on the basis of three major antigenic determinants including somatic (O), Capsular (K), and flagella (H) [60]. The somatic (O) antigen is located at the outer bacterial cell membrane and is heat-stable and forms the oligosaccharide component of the lipopolysaccharide (LPS) of the bacterial cells. More than one O antigen could be expressed by a specific *Salmonella* serotype. The heat-labile H antigens are involved in the activation of host immune responses and are mainly found in the bacterial flagella. Majority of *Salmonella* spp. possessed two different genes, which encode for the flagellar proteins. These bacteria could be diphasic (phase I and II), which means that they possess the unique ability to express only one protein at a time. The phase I H antigens which are responsible for immunological identity could be expressed by some serotypes, whereas phase II antigens are non-specific antigens and could be found in many other serotypes [63]. Finally, the surface K antigens are rarely found among the majority of *Salmonella* serotypes and are heat-sensitive polysaccharides mainly located at the bacterial capsular surface. A subtype of K antigen, the virulence (Vi) antigens are found only in serotypes Paratyphi C, Dublin, and Typhi. “Serovar” a term that is synonymous to serotype has been used commonly in the literature. The subspecies in the naming of a particular *Salmonella* serotype is usually omitted. For instance, *S. enterica* subspecies *enterica* serotype Typhi is normally shortened to *Salmonella* ser. Typhi or *S*. Typhi in literature [60]. So far, over 2500 serotypes have been identified (each having a unique combination of somatic O and flagellar H_1_ and H_2_ antigens), of which >50% of these serotypes belong to the *S. enterica* subspecies. These serotypes account for the majority of *Salmonella* infections in humans [64].

### Morphology, Bacteriological Culture, and Isolation Procedures

The size of *Salmonella* is 0.2-1.5×2-5 μm, and they are facultative anaerobes, rod-shaped, and Gram-negative bacilli of the family Enterobacteriaceae. With the exception of SG and SP, members of the genus *Salmonella* are motile by the means of flagella. Members of this genus have the ability to metabolize nutrients by both respiratory and fermentative pathways referred to as chemoorganotrophic [65]. Majority of the *Salmonella* serovars produce hydrogen sulfide with the exception of few serovars such as *S*. Paratyphi A, and *S*. Choleraesuis. Most members of the genus do not ferment lactose. This important unique property has been used for the development of a variety of selective and differential media for the culture, isolation, and presumptive identification of *Salmonella* [66]. These media included Salmonella-Shigella agar (SS), brilliant green agar (BGA), xylose lysine deoxycholate (XLD) agar, Hektoen enteric (HE) agar, MacConkey agar, lysine iron agar (LIA), and triple sugar iron (TSI) agar [67,68]. Typically, isolation of *Salmonella* from swabs, food, and other environmental samples utilizing the traditional or conventional culture method involves multiple steps of pre-enrichment, selective enrichment, and growth on selective and differential media for the purpose of enhancing the sensitivity of the detection methods [67]. It involves an initial non-selective pre-enrichment of a defined volume of the sample, followed by a selective enrichment step, plating onto selective agars, and then biochemical and serological confirmation of suspect presumptive colonies [1]. In recent years, several regulatory agencies such as association of official analytical chemists, the US food and drug administration (FDA) agency, food safety and inspection service of U.S. Department of Agriculture (USDA), and International Organization for Standardization (ISO) have standardized different approaches of *Salmonella* enrichment utilizing its unique biochemical physical properties. The current ISO standard method, ISO 6579:2002 has been adopted by many *Salmonella* reference centers and is essentially similar to other standard detection methods for *Salmonella* standardized by other regulatory agencies [69].

Briefly, the conventional cultural isolation method consists of pre-enrichment of samples in buffered peptone water (or lactose broth) for recovering of sublethally injured *Salmonella* cells while inhibiting the growth of other competing bacterial flora followed by a selective enrichment in Rappaport-Vassiliadis (Soya base) and Muller-Kauffmann Tetrathionate-Novobiocin containing two or more inhibitory reagents that allow continuous growth of *Salmonella* while suppressing the growth and propagation of other bacteria [68,69]. Subsequently, the incubated selective enrichment broth are streaked on selective media such as SS, BGA, bismuth-sulfite agar, HE agar, and XLD agar. These selective media allow the growth of *Salmonella* organism, while at the same time suppressing the propagation of other bacteria. The colors with the coliforms formed on these media are used for differentiating colonies of *Salmonella* serotypes. For instance, *S*. Typhi on SS appears as colorless colonies with a black center. Typically, *Salmonella* colonies on XLD appear as colorless colonies with black centers, while spherical moist colonies with purple color on BGA [69]. The resulting presumptive colonies isolated on plating media are then incubated in both TSI and LIA followed by tests including urease test and other additional tests for urease negative cultures [1]. Typical *Salmonella* culture conforming with unique reactions is further subjected to biochemical and serological identification tests [1].

### Physical and Biochemical Characteristics

*Salmonella* is non-fastidious because they have the ability to grow and multiply under various environmental conditions outside the living hosts. Although they can grow in the presence of 0.4-4% of sodium chloride, they do not require sodium chloride for growth. Majority of serotypes thrive and grow at a temperature range of 5-47°C with an optimum of 32-35°C. However, some few serotypes can grow at a much wider temperature as low as 2-4°C or as high as 54°C [70]. *Salmonella* is sensitive to heat and is often killed at temperatures of 70°C or above. The pH necessary for the growth of *Salmonella* ranges from 4 to 9, with an optimum range between 6.5 and 7.5. Although *Salmonella* can survive in <0.2 water activity such as in dried foods, they require high water activity of between 0.99 and 0.94 for their survival. The growth of *Salmonella* is completely inhibited at pH <3.8, the water activity of <0.94 and temperatures of <7°C [70]. While almost all serotypes do not produce indole, hydrolyze urea, and deaminate phenylalanine or tryptophan, most serotypes readily reduce nitrate to nitrite, ferment a variety of carbohydrates with acid production, and are negative for Voges–Proskauer reaction [65]. With the exception of *S. enterica* subsp. *arizona*e and *S*. subsp. *diarizonae*, most serotypes utilize arginine, ornithine, decarboxylate lysine and hydrogen sulfide. Similarly, most serotypes utilize citrate with the exception of some few serovars of *S*. Choleraesuis, *S*. Typhi, and *S*. Paratyphi A [65]. While most serovars do not utilize lactose, dulcitol is generally utilized by all serovars with the exceptions of *S. enterica* subsp. *arizonae* (IIIa) and *S. enterica* subsp. *arizonae* (IIIb) [65].

### Pathogenicity and Virulence Factors

The key virulence traits and factors of *S. enterica* such as invasion or intracellular replication inside host’s cells have been approached by various methods such as screening for attenuated mutants, and this has resulted in the identification of many single genes that contribute to the virulence traits at the molecular cellular levels [71]. Many virulence factors have been demonstrated to play variety of roles in the pathogenesis of *Salmonella* infections. These factors included flagella, capsule, plasmids, adhesion systems, and type 3 secretion systems (T3SS) encoded on the *Salmonella* pathogenicity island (SPI)-1 and SPI-2 and other SPIs [72,73]. While other studies revealed that *S. enterica* like many other enteropathogenic bacteria produce a variety of virulence determinants, some of which are part of the adhesion systems including adhesins, invasins, fimbriae, hemagglutinins, exotoxins, and endotoxins [74]. These factors singly or in combination with others allow the *Salmonella* to colonize its host through attaching, invading, surviving, and bypassing the host’s defense mechanisms such as the gastric acidity, gastrointestinal proteases, and defensins as well as aggressins of the intestinal microbiome [14].

*Salmonella* pathogenicity islands (SPIs) are gene clusters located in certain areas of the chromosomes in the bacterial cells that are responsible for encoding the various virulence factors (adhesion, invasion, toxin genes, etc.) [75]. These gene clusters or SPIs can be located on either the plasmid or the chromosomes; compared with the surrounding region they tend to have a variable composition of G/C and are flanked by repeat sequences [76]. The SPIs are characterized to be often associated with transfer RNA (tRNA) and mobile genetic elements such as transposons or phage genes, and they tend to have a base composition entirely different from the core genomes [77]. To date, several SPIs have been reported for different *Salmonella* serovars by different authors with SPI-1 to -5 being the most commonly observed in many serovars of *Salmonella* and others less commonly distributed among the serovars [75,78,79].

In general, the SPIs play different roles in the pathogenesis and virulence of *Salmonella*. Briefly, the SPI-1 is required for the invasion of host cells and induction of macrophage apoptosis, SPI-2 for systemic infections and replication within macrophages, SPI-3 for survival in macrophages and also required for growth of *Salmonella* in low-magnesium environments, SPI-4 for harboring genes responsible for toxin secretion and apoptosis as well as intramacrophage survival, SPI-5 for clustering genes that encode multiple T3SS effector proteins, and SPI-6 has been found in response to external stimuli to transport proteins into the cellular environment or host cells [75,79-84]. In one of these studies [84], the authors reported genetic variations among SPI-1, SPI-3, and SPI-5 while the other two, SPI-2 and SPI-4, were reported to be well conserved among 13 *Salmonella* serovars isolated from various sources including warm-blooded animals (bovine, porcine, avian, and equine), environment, and human patients [84]. Furthermore, the authors also reported that all isolates within the same serovar are identical with respect to the five SPIs tested (SPI-1, SP1-2, SPI-3, SPI-4 and SPI-5) with the exception of those from the ST [84].

Earlier studies reported that most strains of *Salmonella* serovars possessed serotype-specific virulence plasmids. These are plasmid-associated virulence characterized by low-copy-number plasmids (usually one to two copies per cell), and depending on the serovar, its size ranges from 50 to 100 kb [80,85]. In each of these plasmids, there is a *Salmonella* plasmid virulence (spv) locus, where its expression in *Salmonella* organisms has been reported to be important for multiplication of *Salmonella* within the reticuloendothelial system including liver cells and the spleen [80,86]. In addition to serotype-associated virulence plasmids, other plasmids are likely to contribute or confer some resistance observed among *Salmonella* serovars [87]. Other authors reported several different plasmids that are likely responsible for the virulence of serovars such as SH, *S*. Kentucky, and ST [88,89].

Production of both endotoxins and exotoxins has also been attributed to confer pathogenicity among *Salmonella* serovars. The former has been found to elicit a wide range of biological responses, whereas the latter comprising enterotoxins and cytotoxins is associated with killing of the mammalian cells [90]. In one of the studies investigating the production of cytotoxins among *Salmonella* serovars, the authors reported the production of heat-labile trypsin-sensitive cytotoxins with various molecular masses among different serovars including ST (70 kDa), *S*. Choleraesuis (78 kDa), and *S*. Typhi (56 kDa) [90]. In addition, other studies reported two other types of exotoxins namely *Salmonella* enterotoxin and salmolysin encoded respectively by the *stn* and *sly*A genes. These two exotoxins have been identified among serovars Typhi, Enteritidis, and Typhimurium [91]. One study [92] attempted to sequence and clone the salmolysin (product of *sly*A gene) in order to determine its hemolytic property. The authors found out that, the deduced sequence of the salmolysin showed significant homology with regulatory proteins. Therefore, the authors concluded that the hemolytic property of salmolysin could be due to a regulatory event affecting the expression of an *Escherichia coli* hemolysin (HylE) rather than hemolytic activity from the salmolysin itself [92]. Twenty five ST strains recovered from clinical specimens including blood, cerebrospinal fluid (CSF), urine, and feces were studied for markers of virulence [93]. It was found that three of the five isolates from blood, all isolates from both CSF and urine and only two of the fifteen isolates from the feces demonstrated positive fluid accumulation in the rabbit ileal loop. As detected by the latex agglutination and immuno dot blot tests, all the strains positive for the fluid accumulation produced an enterotoxin principle, antigenically related to the cholera family of enterotoxins. Low LD_50_ indicating high virulence was exhibited by all the five isolates from the blood samples, all strains from CSF and one of the two urine strains. This study revealed that some strains of ST are more virulent and produced more enterotoxins as compared to the low virulent strains. The virulent strains invaded the intestinal mucosa and led to extra-intestinal manifestations, whereas the low virulent strains were confined to the intestine and caused mild/moderate gastroenteritis [93].

In an attempt to investigate the production of toxins and their role in the pathogenesis of bloody diarrhea caused by *Shigella* and *Salmonella* from children suffering with bloody diarrhea, human epithelial cells from colon carcinoma (HT-29), Chinese hamster ovary cells (CHO), and kidney fibroblast from rhesus monkey (Vero) were used to detect the cytotoxins [94]. It was found that *Salmonella* strains recovered from the diarrheic children produced cytotoxins and enterotoxins, which could play a role in the intestinal disease. Over 50% of the *Salmonella* strains caused elongation and some strains causing rounding of CHO cells, about 20% of the strains resulted in rounding of HT-29 cells and >60% of the *Salmonella* isolates caused rounding of the Vero [94]. The cytotoxigenicity of different *S. enterica* serovars was also studied on the Madin-Darby Bovine Kidney and Vero cell lines [95]. The serovars tested comprised Typhimurium, Nchanga, Newport, Virchow, Bovismorbificans, Seftenberg, Weltevreden, and Indiana. The authors revealed that all the strains exhibited cytotoxic activity on both the cell lines. However, the cytotoxic activity varied greatly among the serovars and was dose-dependent. Another study [96] reported a positive reaction for enterotoxin production among 76 SE, 3 *S*. Virchow, and 1 *S*. Braenderup strains following screening for enterotoxicigenicity using the CHO, Y1 adrenal, and Vero and HeLa cell tests. In this study, it was found that CHO cells were more sensitive compared to the Vero and Y1 adrenal cells. Overall, this study found that 79 (98.75%) of the investigated strains were producers of enterotoxins as detected from their biological assays. The authors argued that high frequency of enterotoxin production by the *Salmonella* strains might be related to the fact that most *Salmonella* species when present in the gastrointestinal tracts of their hosts are associated with diarrheal disease.

Another important virulence factor for *Salmonella* is HylE protein, which is a product of *hylE* gene [97]. The HylE toxin like many other pore-forming toxins is an important virulence factor among the majority of the bacteria including *Salmonella* [98]. They are important in that they probably play key roles in the pathogenesis of systemic salmonellosis and have been used recently in the sub-serovar level typing [99,100]. Some proteomic studies have demonstrated that production of HylE by *Salmonella* and other enteric bacteria plays a crucial role in the pathogenesis of *S*. Typhi [97]. Another study [101] investigated the HylE patterns of 175 strains of different *S*. *enterica* serovars recovered from different animal sources and places utilizing 11 different blood agar media made with either non-washed horse/sheep erythrocytes or with washed erythrocytes of cattle, sheep, horse, goat, rabbit, guinea pig, and human A, O, and B groups. The findings revealed that all host restricted *S. enterica* serovars, namely SG, *S*. Anatum, *S*. Abortusequi, and *S*. Paratyphi B could be divided into different HylE types based on their inability to produce hemolysis on one or more types of the blood agar utilized. While, other strains of all the zoonotic *Salmonella* serovars induced hemolysis on all the nine types of blood agar made of washed erythrocytes [101]. Further, it was revealed that none of the 175 serovars could produce hemolytic colonies on the blood agar made of non-washed sheep/horse erythrocytes. With the exception of *S*. Abortusequi, the most common HylE pattern observed among all the other studied serovars was HylE type I (lysing all types of washed erythrocytes). In the same study, it was shown that the hemolytic strains of *S*. Abortusequi possessing hemolytic activity against sheep erythrocytes were more invasive but had lesser ability to survive in sheep mononuclear cells as compared to the non-hemolytic strains [101].

To investigate the hemolytic potential of SG strains (94 strains), both phenotypic and genotypic methods including amplification of the HylE gene (*cly*A) and cytolysin gene (*sly*A) were utilized in an attempt to determine their role in HylE production among the studied strains [99]. From this study, the researchers found that the SG strains produced two kinds of hemolysis namely, beneath the colony hemolysis or contact hemolysis (BCH) and clear zone hemolysis (CZH). Hemolysis was observed in blood agar made from the blood of sheep, goat, cattle, buffalo, guinea pig, fowl, horse, and human A, B, AB, and O groups. While *slyA* gene could be amplified uniformly regardless of the hemolytic potentials and patterns of the studied strains, the *clyA* gene was not detected in any of the 94 studied strains. It was suggested from this study that the hemolytic activity – comprising the BCH and CZH – observed among the SG strains might not be due to either *slyA* or *clyA* gene products. Consequently, it was concluded that some genes other than slyA and clyA might be responsible for the hemolytic activity observed in SG strains and the different hemolytic patterns observed on the different blood agar medium could be indicative of the multiplicity of HylEs among the studied SG strains. [99].

Fimbriae play an important role in the pathogenesis of *Salmonella*, and recently it has been shown to represent a source of diversity among *Salmonella* serovars [102,103]. Fimbriae represent the most common adhesion systems, which are differentially expressed and are found in specific patterns among each serovar [104,105]. They mediate adhesion of *Salmonella* to hosts’ cells, food, stainless steel, etc., and have been implicated in a variety of other roles namely biofilm formation, seroconversion, hemagglutination, cellular invasion, and macrophage interactions [73,102,106-110]. The fimbrial systems are normally organized in gene clusters of 4 to 15 genes encoding for structural, assembly and regulatory proteins. With the exception of few fimbriae that are only present in specific serovars, several fimbriae are conserved among *Salmonella* serovars [102]. Until today, the expression, regulation, and roles played by fimbrial genes during the pathogenesis of *Salmonella* infections are poorly understood partly because most *Salmonella* fimbriae are poorly expressed during *in vitro* culture, which further complicates research concerning their regulations and roles [102]. A specific fimbrial gene clusters (FGCs) encodes for the assembly, structural, and sometimes regulatory proteins required for the production of the filamentous adhesive appendage on the bacterial surface [102]. The FGCs are usually composed of 4 to 15 genes [102,106,107]. So far, an average of 12 FGCs by strains was observed in *S. enterica* and in spite of harboring multiple FGCs by the genome of all *Salmonella* strains, only a few have been studied and characterized thus far [102]. Previous studies using mice model investigated the role of ST fimbriae in intestinal cells attachment, persistence in guts, and cecum colonization [111-113]. Furthermore, fimbriae have been demonstrated to be important determinants of host adaptation by *Salmonella* [114].

Flagella located on the cell surface of many bacteria including *Salmonella* have been known to confer pathogenicity besides conferring motility [80]. It is possessed by the majority of *Salmonella* serovars and can be up to 10 normally positioned at random on their cell surface [80]. One of the mechanisms employed by certain *Salmonella* serovars to minimize the host immune response is their ability to display flagellin phase variation, which creates phenotypic heterogeneity of the flagellar antigens [80]. However, the ability and role of flagella (motility and direction of rotation) in the pathogenesis and perhaps their role in adhesion and invasion of mammalian cells still remains unclear [80]. Other virulence factors such as surface polysaccharides may also play role in the pathogenesis of *Salmonella* by allowing the persistence of the bacteria in the intestinal tracts of the hosts [87]. Several studies have identified multiple mutants affecting LPS biosynthesis in *Salmonella* strains isolated from calves and chickens [81,115-117]. One of these studies [115], investigated the virulence in 1-day-old chicks of the LPS *rfbK*, *dksA, hupA, sipC* and *clpB* and *rfaY* transductants, and *ptsC* mutants. The researchers found that all but the *ptsC* and *rfaY* mutants were attenuated for virulence in chickens. While another study [116] on the LPS and ST mutants comprising *rfaK, rfaB, rfaG, rfbP, rfbN, rfbU, rfbH*, and *rfbA* demonstrated that these mutants were unable to colonize the intestines of the calf. The findings from this study suggested the possible role of surface polysaccharides and cell envelope proteins as virulence factors conferring on ST the ability to colonize intestines of the calves. The LPS has been shown to confer on SE the ability to survive in the egg albumen [118].

### Host Specificity and Adaptation

The host specificity of particular pathogenic *Salmonella* depends on the serovar’s ability to adapt to the environment of its hosts. This specific ability to adapt to the host’s environment is regulated by many microbial characteristics, which are responsible for the expression of clinical manifestations in specific host species [119]. Other important determinants included the infectious dose of the particular serovar, animal species infected, host’s age, and immune response. It has been demonstrated that a particular mechanism making a serovar virulent for one particular animal species could make the same serovar less or even avirulent for another animal species [120]. This phenomenon is referred to as “serovar host specificity” or “serovar host adaptation.” For instance, serovars Dublin and Choleraesuis, which are consistently associated with salmonellosis respectively in cattle and pigs [121]. Therefore, host adaptation or specificity is the ability of the particular organism to cause disease in a particular animal population regardless of the degree of pathogenicity it exhibits for a different animal host [119]. An example is the serovar Choleraesuis considered a pig-adapted serovar because it persists in pig populations and not because it causes the severest disease in swine compared to man [121]. It is believed that the process of host adaptation by *S. enterica* serovars involves two mechanisms namely, acquisition of novel genetic elements encoding specific virulence factors and loss of genes [119]. Serovars having host specificity which is dependent on gene deletions included Typhimurium, Enteritidis, Choleraesuis, Gallinarum, Pullorum, Abortusovis, Paratyphi C, and Dublin. Most of the earlier *in vitro* and *in vivo* studies on *Salmonella* host specificity and adaptation were based on the multiplication and survival of *Salmonella* in macrophages from a wide range of animal hosts including humans.

In a study [122] to investigate the differential adaptive evolution of *Salmonella* serovars, a genetic and functional analysis of the mannose-specific type 1 fimbrial adhesin (FimH) was employed. The findings from this study revealed that specific mutant variants of FimH were common in host-adapted (systemically invasive) serovars. Majority of the host-adapted serovars expressed FimH variants with either one of the two phenotypes namely a significantly increased binding to mannose as seen in serovars Typhi, Paratyphi C, Dublin, some of Choleraesuis or complete loss of the mannose-binding activity as demonstrated by serovars Paratyphi B, Choleraesuis, and Gallinarum [122]. Whereas, the low-binding shear-dependent phenotype of the adhesion was found to be preserved in broad host-range (systemically non-invasive) serovars [122]. Recently acquired structural mutations could be responsible for the functional diversification of FimH observed in host-adapted *Salmonella* serovars. Thus, the findings suggested that activation or inactivation of mannose-specific adhesive properties in different systemically invasive serovars reflects their dynamic ability and course of adaptation to the biological environment of their specific hosts. The authors finally demonstrated that mechanisms such as point mutations, the target of positive selection, horizontal gene transfer and genome degradation could be responsible for a differential pathoadaptive evolution of some *Salmonella* serovars [122]. Another study demonstrated that the correlation of some phage types of ST with their hosts and marked host specificity was expressed by the phage types [123]. From this study, however, most of the studied phage types had a broad spectrum of hosts, and this may suggest a phage transfer of virulent genes between hosts eventually leading to host specificity.

Another study [124] assessed *S. enterica* clinical isolates sourced from humans and animals for their virulence capacities and presence of the *Salmonella* virulence plasmid encoding the SpvB actin cytotoxin in mice. The researchers found that all Typhimurium strains derived from animal clinical cases were demonstrated to be virulent also in mice, whereas strains derived from the human salmonellosis patients lacked this ability. It was further revealed that many of the human Typhimurium strains derived from patients with gastroenteritis lacked the *Salmonella* virulence plasmid in contrast to all the animal and human bacteremia strains tested [124]. Furthermore, in contrast to the Typhimurium strains derived from animals phenotypically exhibiting virulent determinants, those derived from man and harboring the *Salmonella* virulence plasmid were avirulent in mice [124]. These findings are suggestive of the fact that *Salmonella* isolates of the same serovar derived from animal salmonellosis are distinctively different from those of human origin. Consequently, these findings suggest that selective pressure within a particular host may give rise to bacterial strain variants exhibiting different pathogenicity determinants and hence varying degree of pathogenicity [119,124].

Another group of researchers [125] from the United Kingdom assessed the factors influencing *Salmonella* host specificity in calves by characterizing the pathogenesis of different serotypes comprising SG, *S*. Dublin, *S*. Choleraesuis, and *S*. Abortusovis. The researchers revealed that through the intravenous route, serotypes Dublin and Choleraesuis were found to be highly and moderately virulent in calves respectively. Both serotypes were found virulent in calves infected orally. In contrast, it was revealed that both serotypes Gallinarum and Abortusovis were avirulent by either intravenous or oral routes [125]. The researchers concluded that these results could be suggesting that initial interactions with the intestinal mucosa by the different studied serovars do not correlate with host specificity, although crucial for the induction of bovine salmonellosis was the persistence of the serovars within tissues and their translocation through an efferent lymphatic system of the calves [125].

Similarly, another group of researchers tested the hypothesis that macrophages are a contributing factor to *Salmonella* host specificity [126]. Although serotype Typhimurium is closely related and shared major virulence loci with the host-specific serovar Typhi that causes disease in humans, Typhi does not cause disease in mice. No significant difference was observed in regard to the survival of the two serovars *in vitro* in mouse macrophage cell lines and primary murine peritoneal and bone marrow-derived macrophages after 24 h. Findings from this study suggest that macrophages were able to distinguish serovar Typhi from Typhimurium when infected *in vivo*; however, no significant difference was observed after 24 h *in vitro*. These results support the fact that the differential killing by macrophages of the two studied serovars may require other intrinsic host factors [126]. In India, research on understanding the problem of host specificity of *S*. Abortusequi was conducted using five isogenic strains including *aroA*, *htrA* and *aroA*-*htrA* deletion mutants, virulence plasmid-cured and wild type parent strains [127]. The strains were tested for invasion, survival and multiplication in macrophages from cattle, goat, buffalo, horse, guinea pig, and murine macrophage-like cells (J-744). With the exception of goat macrophages where invasion rate was comparatively lower, invasion of the different *S*. Abortusequi strains in the different macrophages was not significantly varied. Also revealed was the multiplication of wild type and virulence plasmid cured *S. Abortusequi* in horse macrophages and J-744 cells, suggesting that host specificity and adaptation could be due to the multiplication of *Salmonella* in macrophages. Overall, the findings from the study support the notion that *aroA* and *htrA* genes play crucial roles in macrophages because both the *aroA* and *htrA* deletion mutants failed to survive in cattle and buffalo macrophages as well as in J-744 cells [127].

The degree of host adaptations by *Salmonella* serotypes varies and this affects their pathogenicity for human and animals hosts [128]. Host-restricted serotypes include *S*. Typhi and *S*. Paratyphi (only infect and cause clinical disease in man) and SG and SP (with only poultry as their primary hosts and cause clinical disease in these species). While serotypes such as ST and SE are host-adapted having broad host spectrum and thus, can affect both humans and a wide range of animal species (Table-1). *S*. Typhi and *S*. Paratyphi (Typhoidal strains) are highly adapted to man, and they usually cause severe typhoid syndrome/enteric fever. However, these serotypes are not usually pathogenic to animals [128]. In contrast, serotypes that are highly adapted and have preference for animal hosts may produce mild infections to severe systemic illness in man. For instance, serotypes *S*. Gallinarum and *S*. Abortusovis with poultry and sheep respectively as the primary hosts may cause very mild symptoms in human hosts, whereas *S*. Choleraesuis with swine as the primary host causes severe systemic illness in man. Similarly, *S*. Dublin, which is highly adapted to cattle as the primary host, is responsible for the systemic form of salmonellosis in humans [128]. This serotype causes high mortality in young calves, and other signs include fever, diarrhea, abortion and occasionally death may occur in adult cattle. Among the NTS, serotypes ST and SE (host-adapted) are ubiquitous affecting both man and animals. They generally cause gastroenteritis with less severity than enteric fever. They are also able to asymptomatically colonize chickens. However, studies have shown that these serotypes are capable of causing typhoid-like infections in mice and humans [129].

**Table-1:** Host-specificity and disease syndromes of the representative serotypes [128].

*Salmonella* serogroup/serotype	Hosts	Disease
D/Typhi	Humans	Septicemia, fever
A, B, C/Paratyphi	Humans	Septicemia, fever
B/Typhimurium	Humans, cattle, swine, horses, sheep, poultry, wild rodents	Gastroenteritis, septicemia, fever
D/Enteritidis	Humans, poultry, wild rodents	Gastroenteritis, septicemia, fever
D/Dublin	Cattle, swine, sheep	Gastroenteritis, abortion, septicemia, fever
B/Derby	Birds, swine	Gastroenteritis, septicemia
D/Gallinarum	Poultry	Gastroenteritis, septicemia
B/Abortusovis	Sheep	Septicemia, abortion
B/Abortusequi	Horses	Septicemia, abortion
C/Choleraesuis	Swine	Septicemia, fever

### Transmission of Salmonella

*Salmonella* is ubiquitous and extremely persistent in the dry environment but also in water for periods ranging from days to several months. *S. enterica* serovars have varied hosts and reservoirs and can cause disease in both humans and animals. With the exception of a few serovars that are host-restricted, the majority of *S. enterica* serovars are host-adapted and hence, they can infect and cause disease in a variety of hosts [130]. In farm animals, *Salmonella* can cause clinical disease or subclinical infections in asymptomatic animals refer to as “carriers.” For instance, an earlier study has shown that subclinical infection in hens can persist for >22 weeks [131]. While another study suggested that carrier pigs are an important source of contamination of the environment, other animals in the farms and carcasses at the harvesting stage [132]. These carriers are very important in the perpetuation of *Salmonella* transmission in the farms and environment in that they can shed the organism in their feces continuously and intermittently without manifesting any clinical signs. In a similar way, pets such as dogs and cats have been shown to harbor the organism asymptomatically and thus, could contaminate the environment and other food-producing animals by shedding the bacteria intermittently in their feces [133].

Other important means of transmission include vertical and horizontal transmission [130]. The former involves the transmission of the bacteria from parents to progeny. Vertical transmission is very important especially in poultry related *Salmonella* infection caused by the serovar Enteritidis that has a special affinity for the reproductive system of chickens. In this case, transmission to progeny occurs by transovarian infection when the parent birds have systemic infection leading to infection of the ovary and developing eggs in the oviducts [130]. Another means by which the serovar Enteritidis get access to eggs is by migration from the cloaca to the reproductive organs. Accumulating body of evidence also suggests that *Salmonella* can be transmitted vertically from dam to fetus *in utero* in dairy cattle [134]. On the other hand, horizontal transmission occurs either through the feco–oral or aerogenous routes. Introduction of *Salmonella* into herds can also occur through new purchase and infected pigs; and there is evidence of its spread by fomites, contaminated drinking water, contaminated feeds and dirty feeders, asymptomatic carriers and feces from clinically infected animals in the farm [130].

Pests such as rodents (mice and rats), flies and cockroaches play an important role in the transmission of *Salmonella* from one farm building and facilities to another as well as its perpetuation [130]. Rodents are important vectors and reservoirs of *Salmonella;* they can carry the bacteria in their intestinal tracts asymptomatically without any clinical disease [52]. They have been associated with frequent contamination of feeds, water and stored grains in the farms and can acquire the bacteria mainly from the feces of sick or wild animals in the farm [52,130]. Flies act as mechanical vectors aiding transmission of the bacteria from one farm to another and transmission from cattle to humans has also been documented [135]. Animals in the farm become infected through ingesting *Salmonella*-infected flies. *Salmonella* has been isolated from flies around poultry farms and environments [136-138]. Wild animals such as wild birds and other wildlife are regarded as important reservoirs of *Salmonella* infection [46,47,49,51,52]. They are responsible for the introduction and dissemination of the bacteria into livestock farms through contamination of feed, water or direct environmental contamination [130]. Human trafficking in the farm has been shown to increase the risk of *Salmonella* infection in pigs, chickens, and hens [130]. Another study [139] reported a positive correlation between the entrance of visitors and *Salmonella* prevalence on the farm. Findings from the study suggested that, an entrance of visitors in the farms was associated with higher *Salmonella* prevalence.

### Overview of AMR in Foodborne Pathogens

Antibiotics or antimicrobial agents were discovered around the middle of the 19^th^ century and since then, they have been used for combating the threat posed by pathogenic bacterial agents in both human and animal medicine [140,141]. They are natural, synthetic or semi-synthetic products that are used to inhibit the growth of microorganisms (bacteria) on one end and in the chemotherapy and prevention of infectious diseases in both animals and humans on the other end [142]. Furthermore, farmers use antibiotics extensively either as feed additives or growth promoters to enhance the growth of food animals [143]. Unfortunately, the extensive use or misuse of the antimicrobial agents not only in the treatment of human and animal infections but also as growth promoting agents in livestock production has led to the evolutionary emergence of resistance to one or more of the antimicrobial agents used against the bacterial agents [143,144-148].

AMR is the ability of the microorganisms specifically bacteria to inhibit the agents through different mechanisms from working against them. Over the years, AMR has caused serious public health threat; as the antibiotic agents are no longer effective against the bacterial agents and hence, leading to treatment failures, high mortalities and increase the length of hospitalization among others [145]. Specific bacteria could be resistant to one or more groups of antimicrobial agents. Recently, the European Union (EU) in an effort to reduce the prevalence of antimicrobial-resistant bacteria introduced several actions; one of which is the removal of antimicrobial agents use as growth promoters from all the livestock industry [149]. Furthermore, all countries in the EU have initiated and adopted a new legislation program for surveillance and monitoring of AMR of selected zoonotic and animal pathogens [149].

Among the major contributing factor to the magnitude of the global challenge of AMR is the extensive utilization of antibiotics in food animals [144]. Antibiotics have been used frequently in intensive farming management of food animals such as poultry, pigs and fish for therapeutic or prophylactic purposes for treatment or prevention of bacterial diseases. Furthermore, antibiotics have been extensively used by farmers as growth promoters for enhancing the rapid growth of food animals including poultry and fishes. This further exacerbates the emergence and spread of AMR including MDR [144,150]. The AMR bacteria and antibiotic-resistant genes can cause human infections through entry and transmission at any stage of the food production cycle [144,151,152]. Thus, emergence of AMR bacterial strains along the food chain has posed serious global public health concern because several studies have reported the infection, colonization and contamination of food animals and their products by one or more of the resistant strains such as AMR *Campylobacter* spp., methicillin-resistant *Staphylococcus aureus* (MRSA) and extended-spectrum beta-lactamase Enterobacteriaceae family such as *E. coli*, *Salmonella* spp., and *Shigella* spp. [153-155].

The recent emergence of AMR bacteria such as carbapenem-resistant *Enterobacteriaceae*, colistin-resistant *E. coli* and emerging livestock associated-MRSA has further worsened the current AMR global challenge [154,156]. All these resistant strains have food animals serving as reservoirs and have been associated with high genetic exchanges, virulence mechanisms and adaptability to multiple hosts [153,154,156,157]. These factors can lead to the rapid emergence of novel pathogens that are more resistant, virulent and mobile strains often termed as “superbugs.” The resistant bacterial strains can affect humans through two ways; either following direct contact with infected/or colonized animals or a biological substance such as feces, urine, saliva, or blood of these animals and the other is indirectly along the food chain through consumption of contaminated food or food derived products [151].

### Antimicrobial Resistance (MDR) of Salmonella and Its Surveillance

The first incidence of antibiotic resistance of *Salmonella* was reported in the early 1960s; this was resistance to a single antibiotic namely chloramphenicol [158]. Since then, the isolation frequency of *Salmonella* serotypes resistant to one or more antibiotics has increased globally [159]. This has been related to the misuse, overuse and easy accessibility of antimicrobials in many countries. In the United States, it has been estimated that *Salmonella* causes an estimated 100,000 antimicrobial-resistant infections annually [33]. The overall pattern and trend as well as frequencies of resistance can vary remarkably from one country to another [160]. The U.S’s FDA has recognized the occurrence of AMR in *Salmonella* as well as other bacterial species as a global public health threat since 2003 [15]. Multidrug resistance in *Salmonella* is defined as resistance toward the traditional first-line antibiotics such as ampicillin, chloramphenicol and trimethoprim-sulfamethoxazole [57]. This is a major threat to the public health because a majority of the MDR *Salmonella* infections in humans are acquired by ingestion of contaminated foods of animal origin such as swine, chicken and chicken products such as eggs.

Although the occurrence of NTS in food animals and their susceptibilities to commonly used antimicrobials is poorly understood in developing countries [9], few studies in recent years were conducted to provide epidemiological insights into the ecology, dynamics, environmental drivers and persistence of resistance genes as well as its subsequent transmission along the food chain. For instance, a recent review demonstrated that the prevalence of MDR *Salmonella* is on the rise in the African continent and this may pose difficulty in the treatment of human salmonellosis [54]. In Nigeria, MDR *Salmonella* was reported in Japanese quails suggesting public health risks from direct consumption of these birds or contact with carriers quail birds [20]. Similarly, in Ugandan layer hen farms (n=237), MDR *Salmonella* was identified in 12 (15.4%) of the total isolates recovered and the highest resistance was against ciprofloxacin followed by sulfonamides and sulfamethoxazole/trimethoprim [9]. In Brazil, a 20-year meta-analysis study (1995-2014) was conducted to assess the profile and temporal patterns of AMR of NTS sourced from humans and poultry-related samples [30]. The highest level of resistance was demonstrated against sulfonamides, nalidixic acid and tetracycline by the NTS isolates of poultry origin. Similarly, those of human origins had resistance toward sulfonamides, tetracycline and ampicillin [30]. One study from Taiwan and Thailand [161] isolated and identified *Salmonella* Choleraesuis strains that demonstrated resistance toward cephalosporins and fluoroquinolones. Similarly, another study [148] isolated *Salmonella* from chicken eggs sourced from different marketing channels and poultry farms in Northern India. Moreover, findings from the study revealed that the isolates demonstrated resistance toward bacitracin, colistin, and polymyxin-B. *Salmonella* displaying MDR towards ampicillin and tetracycline was also isolated from table poultry eggs sampled from different sources in Izatnagar, India [162].

Several clones of MDR *Salmonella* have emerged during the late 1990s and early 2000s and since then, their prevalences have expanded globally [15]. Recently, the increasing prevalence of MDR *Salmonella* as well as resistance towards clinically important antimicrobials such as fluoroquinolones and third-generation cephalosporins has become an emerging problem worldwide [26-29]. A recent study from Egypt [163] highlighted the increasing incidence of MDR *S. enterica* in meat and dairy products, which are probably transferred to humans along the food chain subsequently leading to therapeutic failures. In another similar study by Rotimi *et al*. [164] conducted in Kuwait and the United Arab Emirates, the increasing trend of MDR among *Salmonella* isolates was further demonstrated and the rate of resistance toward the third-generation cephalosporins such as ceftriaxone and cefotaxime was reported to have increased by five-fold. Another study from Gondar town of Ethiopia assessed the AMR pattern of *Salmonella* isolates recovered from different sources in the butcher shops [165]. About 28.3% (n=15) of the isolates were MDR with the highest isolation from the meat samples. Ceftriaxone resistance in *Salmonella* remains a serious public health threat because it is commonly used to treat severe *Salmonella* infections especially in children [18]. In an effort to characterize ceftriaxone-resistant *Salmonella* infections in humans from the United States, data reported from the National Antimicrobial Resistance Monitoring System (NARMS) during 1996-2013 were utilized [18]. From this analysis, it was found that 978 (2.9%) of the total 34,100 NTS isolates sourced from humans were ceftriaxone-resistant and many of these (about 40%) were from children younger than 18 years. To identify the diversity of AMR phenotypes among *Salmonella* isolates recovered from integrated commercial broiler farms, retrospective data from the United States NARMS reports were analysed [17]. According to this analysis, 25 AMR phenotypes were observed in the *Salmonella* isolates recovered from two broiler chicken farms with the isolates displaying resistance toward streptomycin alone or in combination with other antibiotics was the most prevalent AMR phenotypes (36.3%) [17]. Another study from chicken carcasses in Myanmar revealed that 72 (52.2%) of the *Salmonella* isolates derived from chicken meat sold at retail markets were MDR [166]. Similarly, a majority (93.75%) of the *Salmonella* isolates recovered from retail chicken and pork in China displayed resistance to multiple antibiotics [157]. In the same study, it was found that MDR was linked only to the *Salmonella* isolates from chickens, whereas those from pork were only resistant to tetracycline. About 7% of the *Salmonella* isolates derived from different sources of poultry farms in the Southeastern United States exhibited resistance to at least one antimicrobials tested [7]. High AMR was observed towards tetracycline, streptomycin and nalidixic acid. Furthermore, a single isolate of *S*. Mbandaka exhibited MDR towards tetracycline, amoxicillin/clavulanic acid and ampicillin [7].

The NARMS was formed two decades ago and it entails an integrated one-health approach in the surveillance and monitoring of AMR in foodborne enteric bacteria from humans, retail meats and food animals [167]. Its primary objectives are to timely identify AMR and provide an updated data on the temporal patterns of antibiotic susceptibilities in *Salmonella* and some selected foodborne enteric bacteria from human and animal populations as well as retail meats. It is the colaboration between the CDC, The U.S. FDA’s center for veterinary medicine and the USDA [167]. In 2007, the NARMS provided an executive summary of the resistance trends among the NTS isolates and the report indicated that 53.9%, 72%, and 43.1% of the isolates respectively from chickens, cattle, and swine exhibited resistance toward at least one antimicrobial tested [168]. In a similar report, NARMS also reported that the most common MDR phenotype among the *Salmonella* isolates was to five antimicrobials namely ampicillin, chloramphenicol, streptomycin, sulfamethoxazole, and tetracyclines (ACSSuT) and this was detected from 1.5%, 4.8%, 16.2%, and 10.9% of the isolates recovered from chickens, turkeys, cattle, and swine respectively [168]. The antibiotic drugs amoxicillin/clavulanic acid, ampicillin, ceftiofur, cefoxitin, chloramphenicol, streptomycin, sulfonamides, and tetracyclines had the highest percentage of resistant *Salmonella* isolates and percentage of the resistant isolates to these drugs has increased since 1997 [168].

In 2013, according to the NARMS’s report data for the U.S., AMR among *Salmonella* strains varied by serotypes [169]. This report highlighted that 3% (61/2178) of the NTS isolates were resistant to nalidixic acid and the common serotypes among the 55 ceftriaxone-resistant isolates were SN, *S*. Dublin, ST, SH and *S*. Infantis. Among these serotypes, Newport, Typhimurium and Heidelberg have been reported to be associated with human infections from food of animal origins [170,171]. Hence, this presented an increasing threat to public health. In another study [172], it was reported that the AMR patterns of over 80% of *Salmonella* strains from both human’s and animal’s sources tested against antimicrobials revealed similar resistance patterns and that the frequently encountered resistance phenotype was resistance to ampicillin, sulfonamides, streptomycin, chloramphenicol and tetracycline (ASSCT). This resistance phenotype was found in 73% and 76% of strains sourced from animal and humans respectively [172]. An earlier study [173] conducted in the Netherlands between 1972 and 1974 screened about 50,000 *Salmonella* isolates recovered from different sources (humans, animals, animal products, sewages, etc.) for resistance against ampicillin, chloramphenicol, kanamycin and tetracyclines. The results of this study indicated that the incidence of resistance to at least one of these antimicrobials tested ranged from 39.2% to 45.6% [173].

There is an increasing frequency of the occurrence of MDR serotypes especially Typhimurium and Newport; and these serotypes along with Heidelberg and Enteritidis have been identified to be associated with human infections from foods of animal origin [170,171]. Recently, surveillance report from the NARMS has shown an increased frequency of the occurrence of extended-spectrum cephalosporin resistance of the serotype Heidelberg isolated from food animals at slaughter, retail meat and humans [174]. In 1984, the ST Definitive Type 104 was first identified in the UK [175] and later isolated from other parts of the world. The emergence of this phage type presented a major threat to public health because it exhibits resistance to five antimicrobials - ACSSuT [176,177]. Compared with infections caused by other susceptible strains, MDR *S. enterica* serotype Typhimurium has been associated with high risk of invasive infection, long duration of hospitalization, longer illness and increased risk of death [15]. For instance, a very unique characteristic of ST serotype is that its genomic element can carry resistance to five antimicrobials namely ampicillin, chloramphenicol, streptomycin, sulfonamides and tetracyclines, which can either spread horizontally to other serotypes or acquire additional resistance determinants from other serotypes [15]. Mobile DNA elements such as integrons and plasmids play an important role in the transmission and dissemination of AMR determinants among *Salmonella* strains [178]. These elements (integrons and plasmids) carry the genes conferring AMR in *Salmonella* and they could be transmitted through process refer to as conjugation [178].

### Public Health Significance of Salmonella

Recently, technological advancements in traveling, globalization and also growth in international trade between many countries in the world have led to the rapid dissemination of foodborne pathogens, contaminants in foodstuffs and other pathogens of potential threat to the human race. Consequently, this lead to an increased perception of the need for adoption of surveillance systems to ensure food safety – identification of foods involved in foodborne outbreaks – due to its economic importance; because the identification of only one contaminated food product may lead to discarding of tonnes of foods resulting in economic losses to the production sector and international trade restrictions [179]. Salmonellosis is one of the most frequently reported foodborne disease outbreaks worldwide but mainly common in developing countries such as India, Asia and Africa [23,54,180]. Salmonellosis poses public health threats due to its high endemicity, difficulty in adopting control measures, and because of its significant morbidity and mortality rates. According to the WHO, *Salmonella* is among pathogens that caused the greatest impact on the human population, and has been associated with outbreaks and sporadic cases of human foodborne diseases worldwide. Poultry and poultry products such as eggs have been frequently reported to be associated with salmonellosis outbreaks and therefore, are generally recognized as primary sources of the disease [181]. Typically, humans become infected through ingestion of foods contaminated with animal feces or cross-contaminated by other sources.

Enteric fever, which is caused by the typhoidal strains *S*. Typhi and *S*. Paratyphi, has been reported endemic in the Southeast and Central Asia, where it causes 200,000 deaths and 22 million illnesses per year [182]. Serovars of NTS are widespread and are commonly associated with specific animals. In the human hosts, they typically cause a self - limiting gastroenteritis with symptoms such as fever, diarrhea, vomiting, and stomach cramps [183]. These symptoms could be accompanied by prolonged fecal shedding of the bacteria for more than a month. Globally, gastroenteritis, the most common form of NTS infection, accounts for about 93.8 million cases and 155,000 deaths per year [34]. Based on a surveillance data for 2001-2005, the frequently isolated serovar responsible for NTS infection worldwide was SE (65%), followed by ST and SN, which respectively accounted for 12% and 4% of the clinical isolates recovered [184]. Similarly, in Asia, Latin America and Europe, SE was the common serotype identified accounting for 38%, 31%, and 87% of the clinical isolates respectively. Whereas, in Africa, both SE and ST were reportedly identified as the common serotypes occurring in 26% and 25% of the recovered clinical isolates [184]. In 2010 alone, the annual costs associated with salmonellosis were estimated at US$2.71 billion for 1.4 million cases [185]. Similarly, in the US, the estimated costs of medical expenses, sick leaves and loss of productivity related to the high incidence of salmonellosis ranged from US$1.3 to US$4.0 billion a year [186].

## Conclusion

The NTS especially serovars Typhimurium, Enteritidis, Heidelberg and Newport have been reported in many outbreaks of human salmonellosis around the globe and these outbreaks have been linked with consumption of *Salmonella*-contaminated foods of animal origins such as poultry and related derived products, pork, fish etc. NTS like many other enteropathogenic bacteria has evolved in utilizing a variety of virulence markers and other cellular machinery to colonize the host by attaching, invading and bypassing the host’s gastrointestinal defense mechanisms. These factors included flagella, capsule, plasmids, adhesion systems and T3SS encoded on the SPI-1 and SPI-2 and other SPIs. These mechanisms are essential for and play crucial roles in the pathogenesis of *Salmonella* infections. Furthermore, the NTS strains have been demonstrated to possess MDR toward the first-line and second-line antimicrobial drugs worldwide. Consequently, an increased frequency of hospitalization, treatment failures, treatment costs, increased morbidity and mortality rates from foodborne salmonellosis cases have been reported worlwide.

## Author’s Contributions

SMJ conceived the review project, design of the review, literature search, wrote the first manuscript draft and edited the final manuscript. All sections of the final review were carefully read, reviewed and approved by the author.
